# Factors Affecting Survival of Common Sandpiper (*Actitis hypoleucos*) Nests along the Semi-Natural Vistula River in Poland

**DOI:** 10.3390/ani14142055

**Published:** 2024-07-13

**Authors:** Marek Elas, Marta Witkowska, Włodzimierz Meissner

**Affiliations:** Ornithology Unit, Department of Vertebrate Ecology and Zoology, Faculty of Biology, University of Gdańsk, Wita Stwosza 59, 80-308 Gdańsk, Poland

**Keywords:** flooding, natural river, predation, riverine habitat, survival rate

## Abstract

**Simple Summary:**

Predation is one of the most important factors shaping birds’ reproductive strategy. For species nesting in the valley of a large lowland river, flooding is also an additional risk, which can significantly reduce nesting success. During this study, we examined what factors affect the nesting survival of the common sandpiper, a small wader species that inhabits waterbodies, especially rivers. Nests located under shrubs were significantly less predated than nests built in grass. Also, locating nests close to water increases the probability of nesting survival. Predation increases as the breeding season progresses, while nests were most vulnerable to flooding at the beginning of the season. The results obtained in this study provide a better understanding of the factors limiting the nesting success of birds breeding in river valleys and can be the basis for planning conservation measures to increase the nesting success of riverine birds.

**Abstract:**

Predation is an important factor limiting bird populations and is usually the main factor influencing nest survival. In riverine habitats, flooding poses an additional significant challenge. Our study aimed to elucidate the influence of nest location and incubation timing on the survival of common sandpiper nests in a large, semi-natural, lowland river. The survey was carried out in central Poland on the Vistula River, in 2014–2015, 2021, and 2023, along two river sections 2 km and 10 km in length. The nest survival rate was 27%, which is twice as low as that reported on small upland rivers, with flooding being an additional factor causing losses on the Vistula River. Our research showed that mammalian and avian predation accounted for 51% of losses and flooding for 49% of losses. The negative impact of floods on nest survival decreased as the breeding season progressed between May and July, while the chances of being depredated increased during the same period. Nests placed under shrubs were less likely predated than nests located in grass. Moreover, locating the nest in proximity to water increased nesting survival and in fact, more nests found in our study were situated close to the water’s edge.

## 1. Introduction

Freshwater and riverine ecosystems are among the most productive ecosystems in the world and support a disproportionately high share of aquatic and terrestrial species relative to the area occupied [1,2,3,4]. Substantial alterations to river systems and anthropogenic pressures have led to a notable decline in both species diversity and habitat integrity [5,6]. Birds inhabiting riverbeds are particularly vulnerable and often show decline in their breeding populations [7]. One of the declining species is the common sandpiper (*Actitis hypoleucos*), a wader species from the family *Scolopacidae* that inhabits waterbodies and rivers of different sizes [8,9]. Its extensive breeding range extends across the temperate zone in the Palearctic from Ireland in the west to Japan in the east [8]. Since 1980, the number of breeding common sandpipers in Europe has declined by over 40% [10]. In Poland and Europe, it is recognized as a species of least concern (LC), although in some countries, it is recognized as an endangered species [11]. Decline in species abundance may be associated with a reduction in the ability of ecosystems to function, and understanding the factors influencing nest success is crucial for the conservation of this declining species [12].

The common sandpiper is a socially monogamous, ground-nesting wader species characterized by biparental incubation: throughout the incubation period, both parents exhibit nest care behaviors directed towards brood protection and predator avoidance [8,13,14]. After hatching, males perform most of the parental care, while females rarely stay with chicks until fledging [15,16], causing them to migrate towards wintering grounds earlier than males [17]. Most of the birds, usually in the same pairs, lay replacement clutches after nest loss [13]. 

The common sandpiper, like other birds nesting in riverine habitats, encounters two prominent factors constraining nesting success: predation and flooding [18,19]. These are primary threats significantly impacting the reproductive output of ground-nesting waders within freshwater environments [20]. Nest inundation is influenced by temporary hydrological conditions, and it can be mitigated by building nests high above water level both on trees by passerines and on elevated places above water level by ground-nesting birds [21,22]. High river flows play a critical role in rejuvenating riverine habitats, providing essential breeding and foraging grounds for both resident [23,24] and migrating birds [25,26]. However, during the breeding season, such flows can cause significant losses to ground-nesting species [27,28]. 

Another response to hydrological fluctuations involves adjusting the timing of nesting, potentially enhancing the probability of nest survival [29]. Common sandpipers in the middle Vistula reduce risk of nest flooding to a greater extent by nesting in more elevated places than by shifting their timing of breeding. Given that the majority of pairs nest at elevations that markedly diminish the risk of nest inundation, this strongly suggests that these birds actively incorporate flood risk into their reproductive strategy [22]. The response of birds to predation can be more complex and may encompass reactions to existing predation pressure as well as an assessment of existing pressure prior to breeding [30]. As the common sandpiper avoids conspicuous behavior around the nest to evade detection by predators [13], we assumed that nest location and nest concealment, as in other ground nesting birds, are important elements in reducing brood losses during the incubation stage [30,31,32]. 

The significance of nest losses caused by predation and flooding varies by location and species depending on the density and the type of predators as well as how birds mitigate predation abundance or weather and hydrological conditions in a given year [28,33,34,35]. Both of these pressures shape birds’ behavior and reproductive strategies [22,35,36]. For breeding populations on small rivers, where common sandpiper nesting success has been studied, flooding and periodic inundation have not been a significant factor shaping nesting success as it is in the valleys of large rivers [37]. This study aims to investigate the nest location features of the common sandpiper that significantly influence nest survival within a semi-natural section of a large lowland river, with a separate examination of the impacts of the two primary causes of the vast majority of breeding failures in riverbed nesting species. 

## 2. Materials and Methods

### 2.1. Study Area

**Figure 1:**
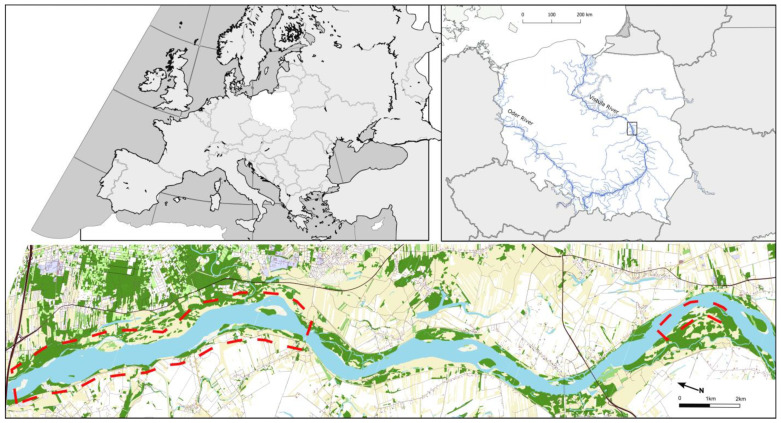
Surveyed sections of Vistula River. The fragments of the surveyed river valley are marked with a red dashed line. Color indications: blue—water; dark green—forests; light green—shrubs or vegetation in the built-up area; yellow—grass vegetation on agriculture land; white—cultivation on agricultural land.

The nest search was carried out on the Vistula River in Poland. In 2014–2015, nest searching was conducted in two nature reserves between 489 and 500 km of the navigational route, whereas in 2021 and 2023, it was conducted along the section of 471–473 km of the navigational route, on a wide permanent river island (Figure 1). Previous pilot studies in the area have shown that the species finds suitable habitats here and nests in high numbers, 35 pairs/10 km [22,38]. The surveyed sections of the river are preserved in a semi-natural state and river regulations such as groins and rip raps are distributed only locally and are not intense [39]. Therefore, natural or semi-natural habitats and fluvial processes are observed in the riverbed, including the dynamic formation and erosion of sand islands, permanent plant islands, and natural steep riverbanks [40]. These habitats are used as breeding sites by common sandpipers, little terns (*Sternula albifrons*), common terns (*Sterna hirundo*), ringed plovers (*Charadrius hiaticula*), little ringed plovers (*Charadrius dubius*), and oystercatchers (*Haematopus ostralegus*), as well as sand martins (*Riparia riparia*) and kingfishers (*Alcedo atthis*), although they do not compete for the same habitat with the common sandpiper, which prefer sites with vegetation [41,42]. The study area is protected as a nature reserve in accordance with Polish law, and as Nature 2000 and IBA sites (Middle Vistula River Valley; PLB 140004, PL083) [42]. Potential predators are birds including the hooded crow (*Corvus cornix*), common raven (*Corvus corax*), and probably also other Corvidae, and mammals like rodents and carnivores such as foxes (*Vulpes vulpes*) or American minks (*Neogale vison*). The surveyed section of the river was not intensively penetrated by people during this study.

The vegetation in the study area has a dynamic character typical for a river valley, with plant communities at different stages of succession: sparsely vegetated sandy islets, pioneer grassland vegetation, shrubs, and forests [43]. The canopy layer is dominated by willow–poplar riparian forest, the shrub layer is dominated by willow *Salix* sp. scrub complex, and the herbaceous layer is dominated by crested pasture and floodplain meadow vegetation [43,44,45].

The landforms are characteristic of systems shaped by fluvial processes, varying on different scales from microhabitats to the scale of an entire river course [46]. Floods occur with varying frequency and magnitude—winter ice-jam flooding, spring floods between March and May due to snow melt and spring rains, and summer floods between June and July after prolonged rainfall. The study area is predominantly flat, but the dynamically occurring processes of sedimentation and ablation generate undulating sections and slopes, allowing for birds to build nests in places that differ in elevation relative to the water level by more than 4 m [22].

### 2.2. Field Studies

The work was carried out from mid-April, when common sandpipers return from winter grounds, to mid-July in 2014–2015, 2021, and 2023. All areas suitable for nesting for birds on the riverbanks and on the islands were surveyed by foot, kayak, or motorboat. Firstly, we mapped territories using a standard method consisting of the observation of singing individuals or courtship pairs [47] adapted to local conditions by adopting the date of control or recording more distinct behavior suggesting an occupied territory or nest [48]. Nests were searched in suitable habitats on riverbanks and islands, mostly based on observations of flushing adults. The nests were controlled at different frequencies depending on their availability and location, but inspections were carried out not less than 2 times during the nesting period, usually at intervals of 3 to 7 days. If the nest was found prior to full laying, the start of incubation was determined on direct observation, based on the assumption that eggs are laid every 1.5 days and incubation starts from the last egg laid [49]. Otherwise, we used the floating test [50], or if the nest was successful, we back-calculated the incubation start date from the date of hatching, assuming that incubation lasts 21 days [49]. 

In 2014–2015, we put Thermochron^®^ iButton data loggers (Maxim Integrated Products, Sunnyvale, CA, USA) into 24 nests, which recorded nest temperature at a frequency of 5 to 20 min over a period of 6 to 20 days. Examining the changes in nest temperature allowed for determining a more accurate date of loss/success than would have resulted from field inspections [51]. Based on the time of the loss, we attributed nest predation to diurnal or nocturnal predation (before or after sunrise and sunset). Successful nests were defined as those in which at least one young was hatched. A situation in which all eggs disappeared from the nest before the date set for hatching or the nest was inundated was considered a loss. Nocturnal predation was attributed to be mammalian, while diurnal can be caused by both birds and mammals [52,53]. We did not take into account nests that were abandoned during the incubation (*N* = 2), as it was impossible to pinpoint what factors caused the abandonment, and the small sample size did not allow for us to analyze those cases as a separate group of nests. Overall, our sample size constituted of 57 nests, out of which 22 were successful, 17 flooded, and 18 predated.

We described the nests using five categorical and two continuous variables (Table 1 and Table 2). Nests were divided into two groups dependent on their location: on the river shore or on the island. The largest permanent island in this part of the river is more than 4 km long and up to 1 km wide and is separated from the actual riverbank by a periodically drying out hallow stream up to 20 m wide. For the purposes of this paper, we considered nests located there as nests on the river shore, as it was easily accessible from the riverbank and there are permanent populations of avian and mammalian predators. We also classified the nests into four groups based on the typical nesting concealment found along the Vistula River: those situated under the shrubs, those located in the grass, nests on sand without any concealment, and nests totally covered by vegetation. However, we were unable to collect sufficient number of nests without any concealment (*N* = 2) and those totally covered (*N* = 2) to include them in the formal analysis. Therefore, those nests were excluded from our dataset. Moreover, we grouped nests into three types according to the topography of their location—on flat, undulated, or sloped terrain. Sloped terrain was classified when the difference between bottom and the top of the slope was 0.75 m or higher and the slope was higher than 45 degrees, undulating when the difference between slope bottom and top was between 0.2 and 0.75 m or slope 15 to 45 degrees, flat terrain, when the difference between the lows and highs of the terrain was lower than 0.2 m. In 2014 and 2015, we observed an unusually high density of breeding birds on one of the islands [38]. To check whether it was an important factor affecting nesting survival, we assembled nests into two groups—semi-colonial and solitary. For semi-colonial nests, we considered nests located on that one small island—where there were, dependent on the year, from 4 to 7 territories of common sandpipers—and nests were located in distance from 2 to 84 one to another [38]. We also modeled the effect of the date of incubation start on nesting survival, as this parameter was proved to be important for the survival of the common sandpiper nests in Vistula River in terms of avoiding inundation [22]. 

Common sandpipers are known to locate their nests in the proximity of water [54], though it was not discovered if it somehow affects the nesting success. Here, we included the distance to the nearest water (i.e., main river channel, oxbow lake, or side channel) as a factor in the nest survival models. All nests are located on the lowest floodplain terrace that is equally inundated in case of flooding. The landform characteristics of fluvial origin often mean that sites located close to the river bank may be higher than sites further away from the river.

### 2.3. Statistical Analysis

We performed nest survival analysis using the package RMark in R version 4.2.2 as an interference for the program MARK [55,56,57]. This analysis allows for us to estimate the probability of the nest surviving an established time interval and including the effect of individual covariates on this parameter. Here, we chose to estimate a daily nest survival rate (DSR), commonly used in studies focused on nest survival. DSR was estimated using data from all nests. This estimate was later used to calculate the overall probability of nest survival from laying full clutch to hatching by raising the obtained DSR value to the power of the number of incubation days (21 in the case of the common sandpiper). However, to test which factors influenced nest survival, we constructed two sets of models for flooded and predated nests separately, since various factors may contribute differently to the probability of failure of both types of nests. To limit the number of interpreted models with possibly spurious variables included in them, for both sets of models, we consider only those variables for which we had an a priori defined reason for inclusion, based on their biological significance and a defined hypothesis that we aimed to test. We used the single-factor models to estimate DSR, as the small sample size available for some levels of variables (Table 1 and Table 2) did not allow for us to construct a more complex model. Following that method, DSR for predated nests was modeled as a function of nest location, nest concealment, topographical location, density of nests, incubation start, or distance to water. DSR for flooded nests was modeled as a function of incubation start or topographical location (Table 1 and Table 2). 

Additionally, in both sets of models, we included a model testing the effect of the year on DSR, to check for variability of this parameter between the studied seasons. All nest survival models assumed constant DSR throughout the nesting period. Models were later ranked based on the Akaike’s information criterion for a small sample size. We drew the inference from the top, most informative models in each set, under the criterion of ΔAIC_C_ < 2 [58].

## 3. Results

The estimated DSR based on the fates of all common sandpiper nests was 0.941 (SE = 0.0097; CI = 0.919–0.957), which with a 21-day-long incubation period resulted in 27% (CI = 17–40%) probability of the nest surviving from laying a full clutch up until hatching. The inclusion of year as a covariate in survival models did not significantly improve the model’s fit in both sets of models for nest survival (Table 3 and Table 4). Predation was the cause of losses in 51% of cases (*N* = 18). In ten cases of losses due to predation, the type of predator or predation timing (day or night) was known. Nocturnal predation attributed to mammals was responsible for 60% (*N* = 6) of predation, while diurnal predation, mammalian or avian, was recorded in 40% (*N* = 4) of cases. Flooding was responsible for the remaining 49% (*N* = 17) of breeding losses.

In the set of models for predated nests, four proposed models were ranked as top models within the provided set, and combined, they accounted for 75.3% of all model weights. Those four models included nest concealment, incubation start, and distance to water as factors used to model DSR, as well as a null, intercept-only model (Table 3). Including topographical location, the density of nests, or nest location as explanatory variables did not improve the models’ fit, pointing to the negligible impact of those factors on DSR. Only one model, containing incubation start as an explanatory variable, was considered as a top model in the set of models for flooded nests and was responsible for 97.4% of all model weights, compared to null, the intercept-only model, and the model including topographical location (Table 4).

**Figure 2:**
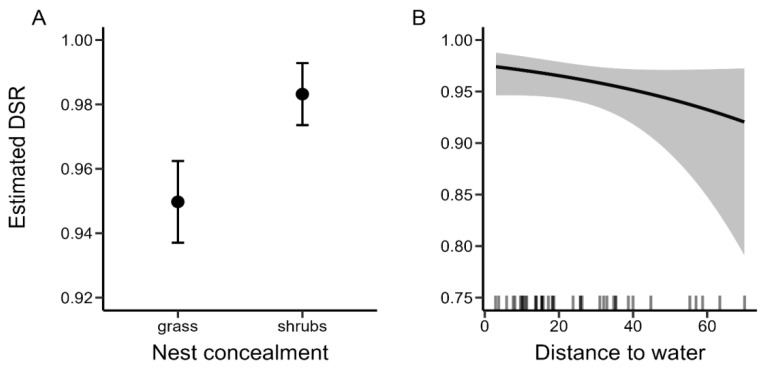
Daily nest survival rates estimated based on top models in the set of models for predated nests for (**A**) two types of nest concealment and (**B**) in relation to the distance of the nest to the nearest water. Dots and black line—estimated DSR values; whiskers—standard errors; grey shaded area—95% confidence interval; bars at the bottom—sample with a given value of the independent variable present in the dataset.

**Figure 3:**
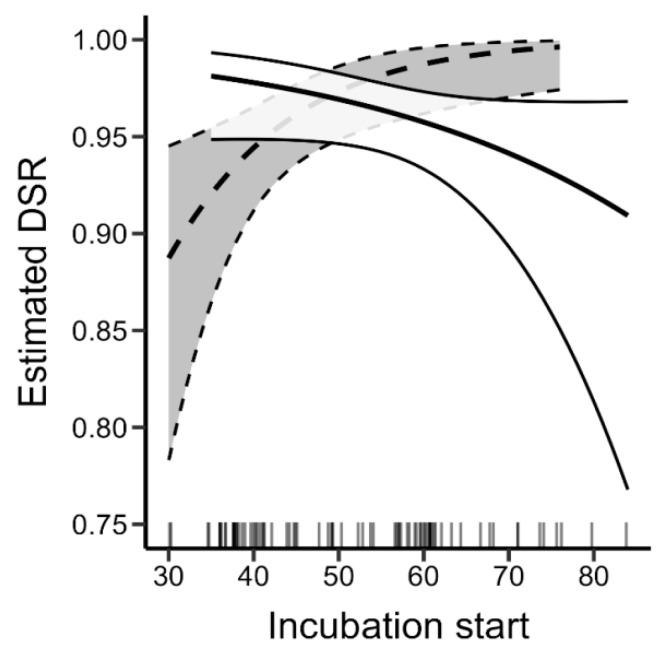
Daily nest survival rate estimated with the top ranking models for flooded nests (dashed line) and predated nests (solid line) in relation to the incubation start. Area around the lines—95% confidence interval, bars at the bottom—sample with a given value of the independent variable present in the dataset.

Nests concealed under the shrubs had higher DSR compared to nests concealed in grass (Figure 2A, Table 5). We observed a decrease in DSR, and therefore increased chances of being predated, in nests in which incubation started later in the season (Figure 3), and in the nests that were located further from the water (Figure 2B, Table 5). However, in the case of those two factors, confidence intervals for the beta estimates were overlapping zero, indicating their weak effect on DSR (Table 5). Additionally, the probability of the nest being flooded decreased in nests with the incubation starting later in the season as the estimated DSR increased with this parameter, indicating a reversed pattern compared to the result of the same model computed for predated nests (Figure 3, Table 5).

## 4. Discussion

The overall probability of nest survival of the common sandpiper on the Vistula River was 27% (DSR = 0.94) and did not significantly differ among years. Nest success on upland rivers in Great Britain ranged from 23% to 50%, estimated using the Mayfield method (50–77% calculated as a percent of territories with success), or from 50 to 89%, calculated as a percent of territories with hatching success [13,37,54,59]. Although the different method of calculation makes comparison difficult, nesting success on the Vistula River was still substantially lower than on upland rivers. The composition of avian predators differed between the upland rivers of Britain and the Vistula valley. In upland rivers, avian predation was mentioned as a major cause of loss. Potential egg predators in studied upland rivers were principally the Eurasian jackdaw (*Corvus monedula*) and Eurasian magpie (*Pica pica*), while on the Vistula River, the hooded crow and common raven were observed the most frequently [13,54]. However, on the Vistula River, mammalian predation is responsible for more than half of the losses.

Although the common sandpiper is a riverine species, according to the available literature, flooding is not a frequently reported cause of nest loss. Inundation represented a minor factor, resulting in the loss of approximately 1–2 nests per year (approximately 1–3% of all nests found) [13,37,54,59]. The impact of floods on nesting success in different sites might be also driven by varied water regimes and times of flooding on rivers, which can change inter-seasonally and in the long term [60,61]. Also, the accessibility of enough elevated terrain can mitigate the flooding rate [19]. It can be thought that the significantly lower nest survival rate compared to populations surveyed on small rivers is due to the more frequent inundation of nests on the Vistula River Valley. 

On the Vistula River, earlier nests of the common sandpiper are more vulnerable to flooding compared to those occurring later in the breeding season. This vulnerability decreases as the breeding season progresses, resulting in fewer nest losses attributed to flooding. These findings align with the results obtained from the analysis of flooding impacts on common sandpiper nest success over a 36-year perspective [22]. Due to the nival river water regime, rainfall floods on the Vistula River are more prevalent during the spring season compared to summer or autumn. However, the exact timing and magnitude of these floods remain fundamentally unpredictable [62]. Consequently, flooding has a higher negative impact on the first, early broods, while in the latter, the impact becomes negligible [22]. Similar to other waders, the common sandpiper lays replacement broods, which is essential when facing a significant level of loss [8,63,64]. Starting breeding early in the season allows for birds to occupy the best territories and provide sufficient time for re-laying [13,63,64]. Although early nesting increases the risk of flooding for the first brood, it can be a trade-off that ultimately leads to increased overall breeding success, as replacement clutches have a much lower risk of flooding. Notably, nesting at an optimal elevation is significantly more effective in reducing flood risk than adjusting the timing of nesting [22]. Hence, the reduced losses due to flooding observed in the latter part of the season are primarily a consequence of hydrological conditions rather than a deliberate strategy by birds to nest later to evade flooding.

In contrast to flooding, nest losses due to predation increased as the breeding season progressed. This rise in predation is typically attributed to changes in habitat conditions [65,66]. Facing predation pressure from both avian and mammalian predators, common sandpipers employ diverse anti-predatory behaviors [52]. The selection of both timing and location for nests represents a trade-off between benefits like nesting in concealment, which can reduce the visibility of the nest, and drawbacks like reducing the possibility of early detection of approaching predators [67]. Furthermore, the majority of predators exhibit opportunistic behavior, and predation pressure may be influenced by the presence of alternative food sources [68,69]. Predation rates are typically density-dependent, meaning that an increase in the cumulative number of active nests can elevate predation pressure [69]. Flooding removes nests from the pool of nests that can be predated just as predated nests are removed from the pool of nests that can be flooded [67]. The recurring 36-year trend of reduced losses due to flooding in the latter part of the breeding season [22] may indicate that a larger pool of nests accessible to predators contributes, at least in part, to the escalating predation rate. Moreover, younger and inexperienced individuals tend to commence breeding later in the season [70,71]. This delayed onset of breeding among inexperienced individuals may contribute to the observed increase in predation rates during the breeding season [72]. It is noteworthy that while the predation rate and flooding rate are in opposition, they do not directly sum up. Thus, from this perspective, the opposite trends are comprehensible, reflecting the intricate interplay between predation and flooding dynamics.

Nest location and concealment represent effective strategies for reducing the probability of nest detection by predators [52,73]. Common sandpiper nests built in the grass or under shrubs were the most abundant along the Vistula River [48]. Although the presence of shrubs as potential perching sites for predators may favor predation by birds and reduce nesting success [74], our study found that nests placed under shrubs exhibited a higher survival rate. For waders that actively protect nests by attacking predators, a critical factor is the ability to detect a predator as quickly as possible and to have the opportunity to chase away the predator. This is easier to achieve in open, woodless, and shrub-free areas [74,75]. The common sandpiper does not actively protect the nest [13]; therefore, although spotting predators from a distance is important, egg crypsis or nest concealment increases the likelihood of their nest’s survival as in other ground-nesting species [31,76]. In the study area, nests were susceptible to predation by diurnal and nocturnal predators, and placing a nest under a shrub can provide additional protection from avian predators, as this kind of physical protection increases their survival rate [76]. 

Common sandpipers nest up to 50 m from the water edge [9], which is consistent with the data we obtained (Table 2). Nests positioned closer to the water had a higher survival probability compared to those located further away. However, it is unclear whether this nesting behavior is a direct response to predation pressure, as predators like the American mink are known to be most active in proximity to water [77]. The advantage of placing nests close to water probably lies in the proximity to feeding grounds, as common sandpipers primarily forage along the water’s edge [78]. Although concealment and visibility from the nest are integral components of the predation prevention strategy, effective vocal communication between incubating birds and sentinel partners also plays a crucial role in the early detection of predators [79]. Moreover, proximity to the water may facilitate more frequent inspections by both the incubation bird and its foraging partner. The complex spatial layout of the valley, with numerous local depressions and elevations, means that horizontal distance does not necessarily correlate with vertical distance relative to the water level. However, the direct cause of higher survival rates for nests situated close to water remains unknown; nesting close to water likely serves not only as a response to the presence of suitable habitat associated with flowing water and periodically flooded riverbanks but also can be a strategy to enhance nesting success through early predator detection.

Although our study showed that under the conditions of a large lowland river, predation and inundation are the main significant factors affecting nest survival, and inundation is more significant than on small rivers on which similar studies have been performed, it should be remembered that we studied only one section of a low-regulated river. On other types of rivers or on regulated rivers, this impact may be different. It is not only the regulation of the river itself that can have an impact on the water regime, but also the degree of transformation in the entire catchment area. Moreover, the small sample size also limits the possibility of inferring the relationship between environmental variables and nesting success. Although we assessed the impact of predation on nest survival, we could not always attribute the loss to a specific predator group. The impact of mammals and birds, as well as individual species from these divisions, therefore requires separate studies.

## 5. Conclusions

Our study suggests that common sandpipers can maximize nest survival probability by nesting close to the water and placing the nest under the shrub. It seems to be a response to mammalian, avian, or both sources of predation and can significantly increase the daily survival rate and mitigate predation risk. On the Vistula River, predation and inundation were two primary factors of nest loss, which appear to be compensatory: later broods face a higher predation rate, while earlier broods are more vulnerable to flooding. Both predation and flooding are selective pressures in populations of ground-nesting birds in riverbeds, shaping the reproductive strategy of ground-nesting riverine species. Our study implies that for ground-nesting riverine species, water regime might be an important factor affecting nesting success, and together with predation needs to be considered when designing active protection measures. Whether breeding populations in the natural or semi-natural ecosystems of large rivers remain sufficiently productive to maintain population numbers despite these multiple threats requires further research, particularly where invasive species such as the American mink or raccoon *(Procyon lotor*) are present [80,81]. 

## Figures and Tables

**Table 1:** Categorical predictors for common sandpiper DSR (daily nest survival rate). Sample size for all levels of each predictor is given separately for nests with different fate (flooded, predated, or successful). na indicates that this predictor was not taken into account in analyzing DSR for a given subset of nests.

		Sample Size		
Variable	Categories	Flooded	Predated	Successful
Nest location	River shore	na	10	13
	Island	na	8	9
Nest concealment	Nest hidden in the grass	na	15	12
	Nest hidden under shrubs	na	3	10
Topographical location	Flat terrain	7	5	6
	Undulating terrain	2	8	7
	Slope	8	5	9
Density of nests	Nests in semi-colony	na	3	7
	Solitary nests	na	15	15

**Table 2:** Continuous predictors, range, means, and standard deviation for common sandpiper DSR (daily nest survival rate). Sample size for each predictor is given separately for nests with different fate (flooded, predated, or successful). na indicates that this predictor was not taken into account in analyzing DSR for a given subset of nests.

				Sample Size		
Variable	Description	Min–Max	Mean ± SD	Flooded	Predated	Successful
Incubation start	Day in the season when incubation started; 1 = 1st April	30–84	51 ± 13	17	18	22
Distance to water	The distance in meters between nest and the closest water (river, oxbow lake, side channel)	3–70	24.5 ± 15.9	na	18	22

**Table 3:** Ranking of daily nest survival models for predated nests. Top ranking models, with the ΔAICc < 2 given in bold. DSR—daily nest survival rate; Null—model with the intercept only; K—number of parameters; AIC_C_—Akaike’s information criterion for small sample size, ΔAICc—difference in AICc between the given model and model with the lowest AICc value; w_i_—Akaike model weight.

Model	K	AIC_C_	ΔAIC_C_	w_i_	Deviance
**DSR~nest concealment**	**2**	**124.05**	**0**	**0.316**	**120.02**
**DSR~incubation start**	**2**	**125.03**	**0.98**	**0.193**	**121.01**
**Null**	**1**	**125.89**	**1.84**	**0.126**	**123.88**
**DSR~distance to water**	**2**	**126.02**	**1.97**	**0.118**	**121.99**
DSR~topographical location	3	126.36	2.31	0.100	120.30
DSR~density of nests	2	126.53	2.48	0.091	122.51
DSR~nest location	2	127.86	3.81	0.047	123.83
DSR~year	4	131.12	7.07	0.009	123.03

**Table 4:** Ranking of daily nest survival models for flooded nests. Top ranking models, with the ΔAIC_C_ < 2 given in bold. DSR—daily nest survival rate; Null—model with the intercept only; K—number of parameters; AIC_C_—Akaike’s information criterion for small sample size; ΔAICc—difference in AICc between the given model and model with the lowest AICc value; w_i_—Akaike model weight.

Model	K	AIC_C_	ΔAIC_C_	w_i_	Deviance
**DSR~incubation start**	**2**	**116.22**	**0**	**0.974**	**112.19**
Null	1	124.30	8.09	0.017	122.30
DSR~year	4	126.72	10.50	0.005	118.62
DSR~topographical location	4	127.28	11.06	0.004	123.03

**Table 5:** Results of the top daily nest survival models for predated and flooded nets. Beta estimates, standard error, and lower (LCI) and upper (UCI) confidence interval limits for given parameters are provided. Parameters with confidence intervals not overlapping 0 are bolded.

Model	Parameter	Estimate	SE	LCI	UCI
Predated nests					
DSR~nest concealment	Intercept	2.369	0.012	2.337	2.421
	**Nest in the grass**	**0.570**	**0.013**	**0.069**	**1.102**
	**Nest under the shrubs**	**1.700**	**0.010**	**1.256**	**2.170**
DSR~incubation start	Intercept	5.137	1.203	2.780	7.494
	Incubation start	−0.034	0.020	−0.073	0.006
DSR~distance to water	Intercept	3.678	0.416	2.862	4.494
	Distance to water	−0.018	0.012	−0.042	0.006
Flooded nests					
DSR~incubation start	Intercept	−0.236	1.172	−2.533	2.060
	Incubation start	0.077	0.028	0.022	0.131

## Data Availability

The data are available upon reasonable request from the corresponding author.
